# Unanswered clinical questions: a survey of specialists and primary care providers*

**DOI:** 10.5195/jmla.2017.101

**Published:** 2017-01

**Authors:** Ellen Brassil, Bridget Gunn, Anant M. Shenoy, Rebecca Blanchard

## Abstract

**Objective:**

With the myriad of cases presented to clinicians every day at our integrated academic health system, clinical questions are bound to arise. Clinicians need to recognize these knowledge gaps and act on them. However, for many reasons, clinicians might not seek answers to these questions. Our goal was to investigate the rationale and process behind these unanswered clinical questions. Subsequently, we explored the use of biomedical information resources among specialists and primary care providers and identified ways to promote more informed clinical decision making.

**Methods:**

We conducted a survey to assess how practitioners identify and respond to information gaps, their background knowledge of search tools and strategies, and their usage of and comfort level with technology.

**Results:**

Most of the 292 respondents encountered clinical questions at least a few times per week. While the vast majority often or always pursued answers, time was the biggest barrier for not following through on questions. Most respondents did not have any formal training in searching databases, were unaware of many digital resources, and indicated a need for resources and services that could be provided at the point of care.

**Conclusions:**

While the reasons for unanswered clinical questions varied, thoughtful review of the responses suggested that a combination of educational strategies, embedded librarian services, and technology applications could help providers pursue answers to their clinical questions, enhance patient safety, and contribute to patient-based, self-directed learning.

## INTRODUCTION

Clinicians often encounter questions in their work setting related to a challenging diagnosis, treatment decisions, or unexpected complications, revealing a perceived knowledge gap. These questions can be self-initiated by clinicians or posed by colleagues or patients. The topic of patient care questions has been well explored in the literature. In 1985, Covell et al. reported that clinical questions during patient care were very common in office practice [1]. Some estimate the frequency of questions to be between one every few outpatient encounters and several questions per patient in an academic medical center [1–3]. A more recent systematic review concluded that clinical questions at the point of care are common, but answers to more than half of these questions are never pursued [4].

Data from the Institute of Medicine estimated that approximately 98,000 deaths were caused by avoidable medical errors in the United States each year [5], and a subsequent study raised the number to 210,000 deaths per year related to preventable harm to hospital patients [6]. Discussions of patient safety efforts typically focus on measures to reduce infection, prevent falls, and inventory surgical equipment, but the danger posed by knowledge gaps should also be considered [7]. Even decades ago, medical educators observed that online searching provided a powerful method of inquiry as well as an alternative to memorizing every bit of relevant new information in an effort to keep up with medical advances and solve new clinical questions [8].

However, answering the clinical question is more complex than undertaking a literature search. Calling to mind the quote by eighteenth century English writer Alexander Pope, “A little knowledge is a dangerous thing,” today’s ease of searching can create a false sense of competence that results in a flawed literature search that ignores critical search techniques and fails to retrieve important articles. Also dangerous is when answers to questions that arise are never pursued, are forgotten, or fall by the wayside.

At best, each unanswered question is a lost learning opportunity and, at worst, it could compromise patient safety. In her book *Patient Safety*, Zipperer presents a compelling case for establishing a critical link between medical error and lack of evidence, information, and knowledge [7]. It is important for health care institutions to devise strategies to alleviate the risk to patient safety caused by clinicians’ knowledge gaps and limited information management skills. Furthermore, the frequency with which answers to clinical questions are not pursued and the potential for concomitant medical errors suggest the need for interventions that ensure timely and accurate answers. To address this issue, the Health Sciences Library at Baystate Health explored how local clinicians, both specialists and primary care providers, responded to clinical questions.

## METHODS

This study was designed and completed in two phases. In phase one, a survey was designed and piloted to a small group of practitioners at Baystate Medical Center. In phase two, the piloted survey was distributed to a wider audience. Both phases of this project were approved as human subjects research by the Baystate Health Institutional Review Board. This article focuses primarily on the second phase of the project.

### Setting

Baystate Medical Center is the academic medical center and the largest of 5 hospitals in the Baystate Health system. Baystate Medical Center serves as the only level-1 trauma care center in western Massachusetts and is a 716-bed facility, offering health care training to nearly 320 residents and fellows, 150 medical students, 1,200 nursing students, and 400 allied health students every year. The Health Sciences Library is fully accessible to all clinicians in the Baystate Health system via the Health Sciences Library website.

### Phase one: survey design and development

To understand the types of clinical questions encountered and the information-seeking behavior of clinicians, two members of our research team (Brassil and Shenoy) facilitated a series of focus groups, including residents, hospital physician attendings, and physician assistants and nurse practitioners (herein, “advanced practitioners”). Data from these focus groups were coded into four themes (identifying and responding to information gaps, background knowledge of search tools and strategies, technology, and interdisciplinary factors), and twenty-seven potential survey items were created from these themes. To ensure that the questions conveyed their intended meanings and were reliable, the research team identified a group of twenty practicing physicians and advanced practitioners from across the institution to pilot the survey. These participants had varying levels of experience and were identified by library staff as having some degree of interest in effective information management. Respondents gave feedback regarding the clarity of each item on the survey. The research team used this information to refine the survey by eliminating ambiguity and biased language as much as possible. After adding a series of demographic questions at the end, the final tool included thirty-five items organized into the four aforementioned themes. The full survey is available in the supplemental appendix.

### Phase two: survey distribution

The final survey was distributed to a total of 1,639 attending physicians, residents, and advanced practitioners at Baystate Medical Center via email. The email invitations assured participants that the survey was optional and anonymous and that those responding could discontinue at any point. The survey remained open for 1 month, with 1 reminder email. Data were collected with an anonymous link created by REDCap [9]. Collected data included a REDCap-generated unique identifier and did not collect Internet protocol (IP) addresses. Data were analyzed with Stata (version 12.0, StataCorp, College Station, TX).

## RESULTS

### Demographics

A total of 292 persons responded to the survey, representing an 18% response rate. However, only 242 persons answered the demographic questions. Most respondents were physician attendings (Table 1), although residents and advanced practitioners also responded to the survey. Most respondents categorized themselves as specialists, reported that more than 80% of their time is spent taking care of patients, and identified themselves as being in practice at least 15 years since obtaining their professional degree.

**Table 1:** Demographics of respondents

	Physicians n=171 (71%)		Residents n=27 (11%)		Advanced practitioners n=44 (18%)		Total n=242 (100%)
	n	%	n	%	n	%	n
Practicing since professional degree							
Less than 5 years	8	5%	18	67%	12	27%	38
5–15 years	51	30%	9	33%	11	25%	71
More than 15 years	111	65%	0	—	21	48%	132
Time per week (on average) taking care of patients							
Less than 50% of time	23	14%	2	7%	3	7%	28
50%–80% of time	35	21%	6	22%	10	23%	51
More than 80% of time	112	66%	19	70%	30	70%	161
Discipline							
Specialist	126	74%	16	59%	34	77%	176
Primary care	45	26%	11	41%	10	23%	66
Location							
Inpatient	39	23%	9	33%	4	9%	52
Ambulatory	64	37%	0	—	25	57%	89
Both	68	40%	18	67%	15	34%	101

### Identifying and responding to information gaps

The majority of providers (84%) encountered clinical questions at least a few times per week. More than half of the respondents (61%) stated that their clinical questions arose from unusual cases, and even more (73%) encountered questions through routine reading. Many clinical questions were initiated by patients (53%) or arose from colleagues and learners (24%), and about half (51%) of respondents reported encountering clinical questions that were important to the situation but were outside the respondents’ area of expertise. Most respondents (57%) indicated a preference to investigate their clinical questions right away, although many also preferred to write down the question (53%) or make mental notes (45%) (Table 2).

**Table 2:** Reported ways that clinicians remember the questions that arise during the workday

	Responses	
	n	%
I investigate the answer right away	167	57
Write them down	155	53
Make a mental note	132	45
Make an electronic note on my phone or tablet	58	20
Send myself an email reminder	51	17
Use EverNote	8	3
Create a file or folder on my computer	8	3
Other	4	1

The vast majority of respondents (88%) claimed that they often or always pursued answers to their clinical questions. When asked to indicate all resources that they consulted when investigating the answers, the most popular resources were an online database (85%), free Internet searching (77%), or personal communication with a colleague (62%). Only 42% used print textbooks to answer their questions. When asked to reflect on a time when they did not pursue the answer to a clinical question, most respondents claimed that time pressures were the biggest barrier or, to a lesser degree, they simply forgot to look up the answer (Table 3). Respondents noted that the largest motivators for pursuing clinical questions were concern for a patient (87%), realization of a knowledge gap (71%), urgency of the question (66%), verification of knowledgebase (63%), and curiosity (60%).

**Table 3:** Reasons for not pursuing a clinical question

	Responses	
	n	%
Time pressures	235	80%
Forgot	137	47%
Searching not integrated	83	28%
Question is low relevance	73	25%
Search tools complicated	31	11%
Unsure how to frame	30	10%
Lack of knowledge	27	9%
Self-conscious	14	5%
Other	6	2%
No requirement to document	5	2%

### Background knowledge of search tools and strategies

When asked which resources were used to answer clinical questions, databases were the major tool of choice (85%). However, 59% of respondents reported no formal training in using search tools such as PubMed. Furthermore, while nearly all respondents were aware of PubMed, only 65% reportedly used it often. Resources that respondents often used were UpToDate (80%), Epocrates (46%), Micromedex (36%), Google Scholar (36%), and Cochrane (35%). Other responses pointed to an ignorance of various search tools, as respondents were not at all familiar with many licensed products (e.g., Natural Standard, 95%; Access Medicine, 81%; Scopus, 78%; and ClinicalKey, 73%). In a specific question regarding clinical decision support (CDS) tools, only 15% of respondents indicated that they have used this type of tool, and just over half (55%) indicated that they “do not know what a CDS tool is.”

Approximately half of the respondents (51%) agreed or strongly agreed that they were familiar with ways to refine search queries, such as using subheadings or search filters. However, when presented with a list of filters typically used to refine results—such as publication date, language, demographic variables, and/or systematic reviews—21% of respondents reported using none of them, and another 25% reportedly used only 1 type of filter. A little over half of the respondents (58%) had formal training on the critical appraisal of medical literature.

**Figure 1:**
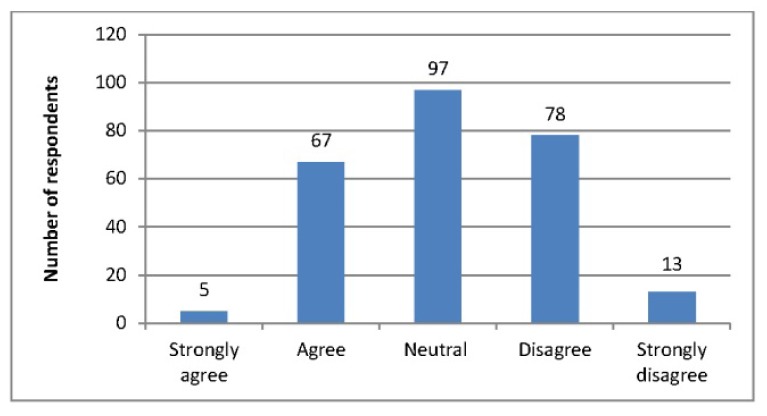
I generally assume that the most current article supersedes earlier literature on a given topic

**Figure 2:**
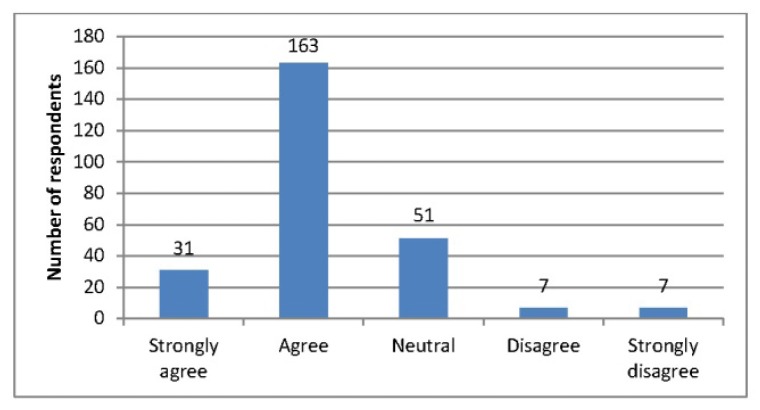
I consider the authority of the articles that I read

Respondents were also asked about how they searched literature and how they appraised the articles that they found. When asked to indicate which factors they considered when searching the literature, the most popular responses included: the relevance of the information to the patient situation (85%), the date of the publication (73%), the type of study (65%), and a critical appraisal of the source (50%). Respondents indicated being attracted to certain journals most often because of easy accessibility (76%) and recognition of the journal as a “go to” or core journal for the specialty (66%). Respondents were mixed in the extent to which they agreed that the most current article superseded earlier literature on a given topic during a search (Figure 1). However, a clear majority of respondents agreed that they considered the authority of the articles they read (Figure 2).

### Technology

The vast majority of respondents (97%) agreed at least somewhat with the statement that the convenience of new technology was worth the occasional challenges. Furthermore, most respondents (93%) owned a mobile device, and most respondents (79%) used mobile devices to access clinical information and/or had clinical apps installed on their devices (72%). To broaden their information base or stay apprised of the latest literature, few respondents indicated that they used email discussion lists (16%), automatic search updates (18%), or electronic tables of contents (21%), whereas more respondents indicated that they attended conferences (54%), read reviews (53%), or performed literature searches as needed (29%).

### Interdisciplinary factors

In this section of the survey, respondents were asked if and how they consulted with experts outside their fields to pursue answers to clinical questions. When asked what type of information they sought from pharmacists, the most common answers were drug dosing (57%) and drug interactions (48%). Over half of the respondents (67%) indicated that they had asked for help from a Baystate Health librarian, most often when they could not find the information needed on their own (45%) or when they were unsure about some of the database search tools (28%).

More than half of the respondents (66%) somewhat or strongly agreed that having a librarian embedded in the clinical work setting to search the literature would help to answer clinical questions. Most respondents (70%) indicated that they would utilize a live chat or info button on their desktop or mobile devices to forward clinical questions to a librarian.

When asked which library resources best supported their information needs, the majority of respondents chose literature searches (64%) and the ability to obtain a specific article (63%). To a lesser extent, respondents valued librarians’ abilities to access core textbooks or electronic books (36%) or to offer assistance with mobile devices and apps (30%), automatic search updates (17%), electronic tables of contents (17%), and email discussion lists (10%). A minority of respondents (9%) reported not using the library at all.

## DISCUSSION

The goal of our survey was to build on the existing literature regarding unanswered clinical questions, while gathering information to help characterize future interventions that might help clinicians answer these questions. Like prior studies [1, 4], our results revealed that clinical questions requiring investigation are frequent, with the majority of clinicians encountering these types of questions at least a few times per week. These questions were common whether a respondent was an attending physician, a resident or fellow, or an advanced practitioner. However, in contrast to prior studies in which a large number of these questions were never followed up [4, 10], most of our respondents indicated that they often or always pursued answers to their clinical questions. To this end, we propose three interventions designed to address the needs of our clinicians.

First, our clinicians relied most heavily on online databases, Internet searching, and consultation with colleagues when investigating answers, consistent with findings from recent studies [11, 12]. However, our survey demonstrated a lack of formal training among clinicians in searching these databases. Furthermore, many practitioners in this study previously reached out to librarians when they could not find information on their own, were not confident in their searching abilities, or did not have time to pursue the answer. These factors suggest a need for formal training in this area, as our providers do encounter questions that require more advanced searching skills. While each information source varies in its search interface and features, and while new applications and tools are continuously developed and upgraded, the same basic evidence-based medicine and search skills are transferable across resources. Therefore, even increased formal training in one database, such as PubMed, can lead to increased familiarity and proficiency in using search concepts that are applicable to other resources.

Second, while at least one intervention should address formal training, our study results also suggest a favorable response to embedded librarians in the clinical setting. Our next suggestion is to deploy a librarian into high-need clinical areas, which could have a positive impact on information-seeking behavior and patient safety outcomes. This is in keeping with the analogy made by Byrd between librarians and pharmacists as consultants to the health provider at the point of care [13]. Lack of time and forgetting the question were the most common reasons for not pursuing answers among our clinicians, consistent with findings from previous studies [1, 12, 14–16]. This highlights the need for a potential intervention at the moment the question arises, which could be partially addressed by an embedded librarian model.

Third, time pressures were the most popular reason why clinical questions were left unanswered. Especially in acute care inpatient settings where there are not many pauses between patient encounters, our clinicians would benefit from a mechanism to track questions that arise during their workflow so that they can pursue answers afterward. An info button in the electronic health record system to enlist a librarian’s help with a question could fulfill this need and would approximate the embedded librarian concept by integrating support into the workflow when most questions occur and when many practitioners prefer they be addressed [15]. This type of immediate access could also be achieved with the use of mobile technology tools. Our study results indicate that our clinicians are receptive to new technology and that most already use mobile devices to access clinical information, opening the door for enhanced mobile interventions such as a search request app, current awareness alerts, and other clinical tools that could be readily accessed at the point of care when the need arises.

Unlike prior studies that largely focused on outpatient and primary care physicians [4], many of our responses came from clinicians who spent some time in the inpatient setting and are specialists. However, there were no differences in how different populations of health care providers responded to our survey questions, from tracking clinical questions to pursuing the answers. Based on these results, any intervention to support the capture and investigation of clinical questions could be universally beneficial and may not need to be tailored to individual provider types. It should be noted that our study did not further parse out differences between subspecialists, and so we recommend this area for future research. Another limitation of our study is the relatively small number of resident responses, which prevented our ability to perform any sub-analysis on this group. While this would be valuable information, our data do help us understand the habits of their teachers: physician attendings and advanced practitioners.

Lastly, our survey respondents included advanced practitioners who were at various stages in their careers but spent the majority of their time seeing patients. In the era of physician shortage, there has been a push for increasing the role of advanced practitioners [17, 18]. Along with this push have been voices of caution that this evolution of the advanced practitioner role will require better educational infrastructure [17]. Because our advanced practitioners mirrored their physician colleagues in regard to how and why clinical questions were or were not pursued, we believe that similar interventions can be used to address clinical questions for both groups.

Since there is no single, favorite approach to capturing the clinical question, libraries and clinical training programs should explore multiple, interactive strategies and interventions, similar to those identified in this article, to help providers access clinical information at the point of care. While electronic resources can offer convenience and save time, new information technology alone is insufficient for strengthening a culture of safety. It is likely that a combination of targeted educational strategies, embedded librarian services, and technology applications would help practitioners recognize a clinical question and follow through to its resolution—thus enabling patient safety efforts and lifelong learning.

## SUPPLEMENTAL FILE

Appendix Clinical questions survey
