# Supplementary material for: Unanswered clinical questions: a survey of specialists and primary care providers
Source: J Med Libr Assoc. 2017 Jan;105(1):4–11. doi: 10.5195/jmla.2017.101 (PMC5234458; doi:10.5195/jmla.2017.101)
Supplement: Appendix [file jmla_jan17_brassil_app.pdf]

JMLA Journal of the Medical Library Association

SC
Supplemental content to
J Med Libr Assoc. Jan;105(1): dx.doi.org/10.5195/jmla.2017.101
www.mlanet.org
© Brassil, Gunn, Shenoy, Blanchard 2017

Unanswered clinical questions: a survey of specialists and primary care providers

Ellen Brassil, MSLS, MAT, AHIP; Bridget Gunn, MSLS, MS, AHIP; Anant M. Shenoy, MD; Rebecca Blanchard, PhD

APPENDIX
Clinical questions survey

This survey includes some questions that may be sensitive or personal. You are free to skip any question for any reason. There is a minimal risk in participating in this survey, but there is always the risk of loss of confidentiality, even though responses are anonymous. We will not record any personal information about you. You may not benefit directly from this study, but the information gathered from this survey may help in improving library resources.

By agreeing to continue with this survey you are consenting to participate in this research study with the understanding that you are free to withdraw at any time. If at any time you wish to discontinue your participation, you may exit the survey and it will not result in any penalty or loss of benefits to which you are otherwise entitled. Your participation or refusal in this survey will not affect your employment or performance evaluation.

Thank you in advance for taking the time to complete this survey.

Section 1: Identifying and responding to information gaps

1. Generally, how often do you encounter a clinical question that requires further investigation?
   - o Several times a day
   - o Once a day
   - o A few times per week
   - o Once a week
   - o Once a month
   - o Less than once a month
2. How do you remember the questions that arise during your workday? (check all that apply)
   - o Write them down
   - o Make a mental note
   - o Send myself an email reminder
   - o Use EverNote
   - o Create a file or folder on my computer
   - o Make an electronic note on my phone or tablet
   - o I investigate the answer right away
   - o Other

   What other method(s) do you use to remember your clinical questions? ____________________

Supplemental content to
J Med Libr Assoc. Jan;105(1): dx.doi.org/10.5195/jmla.2017.101
www.mlanet.org
© Brassil, Gunn, Shenoy, Blanchard 2017

3. Clinical questions typically arise from: (check all that apply)
   - o Patient-initiated
   - o Colleagues and learners
   - o My routine reading of the literature in my field
   - o Unusual clinical cases
   - o Diagnostic findings (lab, radiology, pathology, etc.)
   - o Medication and dosing
   - o Other

   From what other source(s) do clinical questions arise? ____________________

4. How often do you encounter clinical questions that are important to the clinical situation but are outside your area of expertise?
   - o Always
   - o Often
   - o Sometimes
   - o Rarely
   - o Never

5. How often do you investigate answers to your clinical questions?
   - o Always
   - o Often
   - o Sometimes
   - o Rarely
   - o Never

6. What prompts you to look up information on a clinical question? (check all that apply)
   - o Urgency of question
   - o Concern for individual patient
   - o Pure curiosity
   - o Repeat question that has arisen previously
   - o Upcoming presentation or teaching opportunity
   - o Realization that I have a knowledge gap
   - o Verify that my knowledgebase is still accurate
   - o Other

   What else prompts you to look up information? ____________________

7. The most common reasons for *not* pursuing a clinical question are: (check all that apply)
   - o Time pressures
   - o Forgot about the question
   - o Self-conscious about exposing ignorance or knowledge gap
   - o There is no requirement that I document any sources consulted
   - o Question is of little clinical relevance
   - o Lack knowledge about where to look for the answer

SC
Supplemental content to
J Med Libr Assoc. Jan;105(1): dx.doi.org/10.5195/jmla.2017.101
www.mlanet.org
© Brassil, Gunn, Shenoy, Blanchard 2017

- o Unsure how to frame the question/search
- o Search tools are too complicated
- o Searching is not integrated easily with my clinical workflow
- o Other

What other reason(s) do you have for not pursuing a clinical question? ______________________________

8. When deciding to pursue a clinical question, which resources do you use? (check all that apply)
   - o Ask a colleague
   - o Print media (e.g., textbook, journal, or other reference text)
   - o Online subscription-based media (e.g., electronic textbook, journal, or other reference text)
   - o Free Internet search engines and websites (e.g., Google, Bing, Wikipedia)
   - o Online database or point-of-care tool (e.g., PubMed, Ovid, Cochrane, UpToDate)
   - o Other

   What other resource(s) do you use to find clinical information? ______________________________

Section 2: Background knowledge of search tools and strategies

9. Have you had any formal training on the critical appraisal of medical literature?
   - o Yes
   - o No

   If yes, please describe briefly: ______________________________
10. Have you had any formal training in using search tools such as PubMed?
    - o Yes
    - o No

    If yes, please describe briefly: ______________________________
11. Have you used clinical decision support (CDS) tools to help take care of patients?
    - o Yes
    - o No
    - o I do not know what a CDS tool is

    If yes, which CDS tool(s) have you used? ______________________________

JMLA Journal of the Medical Library Association

SC
Supplemental content to
J Med Libr Assoc. Jan;105(1): dx.doi.org/10.5195/jmla.2017.101
www.mlanet.org
© Brassil, Gunn, Shenoy, Blanchard 2017

How familiar are you with the following resources?

	Aware of this resource and have used often at BH or elsewhere	Aware of this resource but have not used very often	Not familiar with this resource
Access Medicine	o	o	o
BMJ Best Practice	o	o	o
Clin-eguide	o	o	o
ClinicalKey	o	o	o
Cochrane Library	o	o	o
Control of Communicable Diseases Manual	o	o	o
DynaMed	o	o	o
Epocrates	o	o	o
Evernote	o	o	o
Google Scholar	o	o	o
Isabel	o	o	o
Johns Hopkins	o	o	o
Journal Watch	o	o	o
Micromedex	o	o	o
Natural Standard	o	o	o
Nelson's	o	o	o
Professional email discussion lists	o	o	o
OvidSP	o	o	o
PubMed	o	o	o
Red Book Online	o	o	o
Scopus	o	o	o
Semantic Qualifiers	o	o	o
UpToDate	o	o	o
VisualDx	o	o	o

13. When searching for information on clinical questions, I consider the following factors: (check all that apply)

- o Relevance of information to patient situation
- o Date of publication
- o Variety of opinions
- o Author's reputation/expertise
- o Historical background/aspects or context of topic
- o Critical appraisal of the source
- o Type of study (e.g., systematic review)
- o Need for exhaustive search
- o Other

SC
Supplemental content to
J Med Libr Assoc. Jan;105(1): dx.doi.org/10.5195/jmla.2017.101
www.mlanet.org
© Brassil, Gunn, Shenoy, Blanchard 2017

14. I am attracted to certain journals because of the following factors: (check all that apply)
    - o Easy accessibility
    - o Prestige
    - o Impact factor
    - o A "go to" or core journal for my specialty
    - o Frequency of publication of issues
    - o Ancillary features (e.g., podcasts, continuing medical education [CME], clinical cases)
    - o Integration with mobile devices
    - o Other

    What other journal criteria do you consider important? ________________________________

15. I generally assume that the most current article supersedes earlier literature on a given topic.
    - o Strongly disagree
    - o Disagree
    - o Neutral
    - o Agree
    - o Strongly agree

16. I consider the authority of the articles that I read.
    - o Strongly disagree
    - o Disagree
    - o Neutral
    - o Agree
    - o Strongly agree

17. I am generally familiar with ways to refine clinical queries using features like subheadings and filters when searching online databases.
    - o Strongly disagree
    - o Disagree
    - o Neutral
    - o Agree
    - o Strongly agree

18. When doing a literature search (e.g., in PubMed), I often refine my search results using: (check all that apply)
    - o Systematic reviews filter
    - o Age and other demographic variables
    - o Language of publication
    - o Publication date
    - o Indicate that a specific subject/concept must be a main focus of the articles
    - o Other

    How else do you refine your search results? ________________________________

© Brassil, Gunn, Shenoy, Blanchard 2017

Section 3: Technology

19. I own a mobile device such as a tablet or smart phone.
    - o Yes
    - o No
20. I use my mobile device to access clinical information.
    - o Yes
    - o No
21. I have added mobile apps to my device that allow me to access or search for clinical information.
    - o Yes
    - o No
22. In general, new technologies can be counter-productive, creating more challenges to finding clinical answers.
    - o Strongly disagree
    - o Disagree
    - o Neutral
    - o Somewhat agree
    - o Agree
    - o Strongly agree
23. The conveniences of new technology often make the challenges a worthwhile tradeoff.
    - o Strongly disagree
    - o Disagree
    - o Neutral
    - o Somewhat agree
    - o Agree
    - o Strongly agree

Section 4: Interdisciplinary factors

24. I sometimes seek the expertise of the pharmacist for the following types of questions: (check all that apply)
    - o Dosing
    - o Drug interactions
    - o New drugs
    - o Adverse effects of drugs
    - o Contraindications of drugs
    - o I never contact a pharmacist
    - o Other

    For what other reason(s) do you contact the pharmacist? ______________________________
25. In an effort to broaden my information base, stay apprised of the latest literature, and/or meet my specialty's maintenance of certification (MOC) process, I commonly use the following strategies: (check all that apply)
    - o Automatic search updates on favorite topics (e.g., RSS feeds)
    - o Automatic tables of contents sent to my email
    - o Conferences
    - o Email discussion lists
    - o Regular literature searches
    - o Read article reviews (e.g., Journal Watch)
    - o Follow certain experts/authors for new publications
    - o Other

    What other strategy/strategies do you use? ______________________________

SC
Supplemental content to
J Med Libr Assoc. Jan;105(1): dx.doi.org/10.5195/jmla.2017.101
www.mlanet.org
© Brassil, Gunn, Shenoy, Blanchard 2017

26. Having a librarian embedded in the clinical work setting (i.e., during rounds or meetings) to do literature searches would help to capture clinical questions that might otherwise fall by the wayside.
    - o Strongly disagree
    - o Disagree
    - o Neutral
    - o Somewhat agree
    - o Agree
    - o Strongly agree

27. I would use a live chat or info button located on my desktop PC or mobile device to forward clinical questions to a librarian.
    - o Yes
    - o No

28. Which of the following library resources best supports your information needs? (check all that apply)
    - o Literature searching
    - o Setting up automatic updates of earlier searches
    - o Obtaining specific articles that I need
    - o Accessing email discussion lists
    - o Receiving tables of contents of selected journals in my email
    - o Conference information
    - o Core textbooks and e-books
    - o Assistance with mobile devices and apps
    - o I do not use library resources
    - o Other

    What other library resource(s) support your information needs? ______________________

29. I have asked for help from a Baystate librarian.
    - o Yes
    - o No

30. I tend to contact a librarian when: (check all that apply)
    - o I cannot find the information on my own
    - o I need to conduct an exhaustive search and don't want to miss anything
    - o I need to conduct an exhaustive search using database(s) that I am not familiar with
    - o I am unsure about some of the database search tools that might be helpful
    - o I have a question but don't have time to pursue it
    - o I never contact a librarian
    - o Other

    For what other reason(s) do you contact a librarian? ______________________

SC
Supplemental content to
J Med Libr Assoc. Jan;105(1): dx.doi.org/10.5195/jmla.2017.101
www.mlanet.org
© Brassil, Gunn, Shenoy, Blanchard 2017

Demographics

69. What is your title?
    - o Attending (MD, DO)
    - o Resident/fellow
    - o Advanced practitioner (PA, NP)
70. Are you a primary care provider or a specialist?
    - o Primary care
    - o Specialist
71. What is your primary location of practice?
    - o Inpatient
    - o Ambulatory
    - o Both
72. How long have you been practicing since medical school or since PA/NP degree program?
    - o Less than 5 years
    - o 5–15 years
    - o More than 15 years
73. In a typical work week, what is the approximate amount of time you spend taking care of patients?
    - o Less than 50%
    - o 50%–80%
    - o More than 80%
